# Concurrent Acute Appendicitis and Type III Appendiceal Intussusception: A Case Report

**DOI:** 10.7759/cureus.27310

**Published:** 2022-07-26

**Authors:** Christopher G Hurtado, Lily Chen, Teerin Meckmongkol

**Affiliations:** 1 Pediatric Surgery, University of Central Florida College of Medicine, Orlando, USA; 2 Pediatric Surgery, Nemours Children’s Hospital, Orlando, USA

**Keywords:** pediatric laparoscopic surgery, pediatric intussusception, partial cecectomy, appendicitis, appendiceal intussusception

## Abstract

Appendiceal intussusception is exceedingly rare. Although there are few case reports of concurrent ileocecal intussusception and acute appendicitis, to our knowledge, this is the first reported case of concurrent Type III appendiceal intussusception and acute appendicitis. We present the case of an 11-year-old male who underwent appendectomy with partial cecectomy for a Type III appendiceal intussusception with concurrent acute appendicitis.

## Introduction

Appendiceal intussusceptions are exceedingly rare with an estimated incidence of 0.01% [1]. Initial reports were more common in children; however, a more recent study found increased rates in adults [1,2]. Interestingly, incidence broken down by age and gender reveals that appendiceal intussusception is more common in females in the adult population, while it is more common among males in the pediatric population.

Appendiceal intussusceptions are rarely diagnosed preoperatively, with one review reporting only 12 cases described in the medical literature [3]. Preoperative diagnosis is challenging and typically requires multiple diagnostic imagining studies, including abdominal ultrasound, barium contrast enema, and abdominal computed tomography (CT). However, even with all these imaging studies, dilated bowel loops may hide sonographic signs of an intussuscepted appendix [4].

Non-surgical reduction of appendiceal intussusception with barium, water-soluble contrast, and air enema has been unsuccessfully attempted in the pediatric population [4-6].

## Case presentation

An 11-year-old male presented with a one-week history of worsening generalized abdominal pain that subsequently localized to the right lower quadrant. Physical examination was pertinent for a soft, non-distended abdomen which was tender to palpation in the right lower quadrant with a positive Rovsing sign and obturator sign. Incidentally, a cardiac murmur was also noted on the examination. Workup revealed leukocytosis with a white blood cell count of 18,000, and coronavirus disease 2019 (COVID-19) polymerase chain reaction (PCR) testing was negative. He underwent a CT of the abdomen/pelvis that demonstrated a dilated fluid-filled appendix visualized in the right lower quadrant measuring up to 12 mm in diameter with mucosal hyperenhancement consistent with uncomplicated appendicitis. There were no appendicoliths or signs suggestive of perforation or abscess formation. Cardiology was consulted and the patient was cleared for surgery. The patient received ceftriaxone and metronidazole per our institution’s appendicitis management protocol.

**Figure 1:**
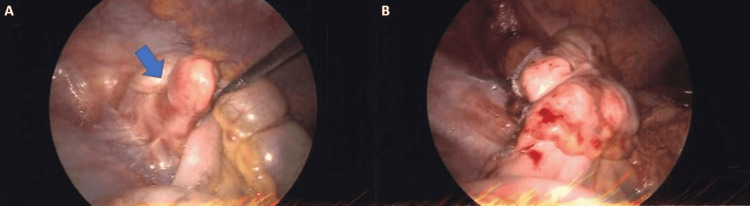
Intraoperative visualization of the appendix. (A) Intussusception of the appendiceal base (blue arrow) visualized during laparoscopy. (B) Portion of the cecum is shown post-laparoscopic appendectomy with partial cecectomy.

The patient was taken to the operating room for routine laparoscopic appendectomy for acute appendicitis. As we attempted to identify the appendix and define the anatomy, we encountered the base of the appendix intussuscepted into the cecum (Figure 1). Multiple varied attempts were employed to reduce the intussuscepted appendix; however, they were unsuccessful and aborted due to concerns of serosal injury to the cecum, as well as traumatically fracturing the inflamed appendix. We were able to determine that the appendectomy would be feasible by sacrificing a small segment of the lateral distal cecum while preserving the medial portion of the cecum and the ileocecal valve. Careful attention was given to ensure no residual appendiceal stump was left behind because residual appendiceal stumps have been found to cause recurrent intussusception or can lead to appendicitis recurrence. The appendix and the intussuscepted portion found in the cecum were sent to pathology. The final pathology report showed focal transmural acute inflammation of the appendix with the proximal appendix showing edema and serosal fibrosis consistent with intussusception. The patient tolerated the procedure well and the postoperative course was uneventful. The patient was discharged on postoperative day one. The patient did well in the follow-up visit with no complications.

## Discussion

**Figure 2:**
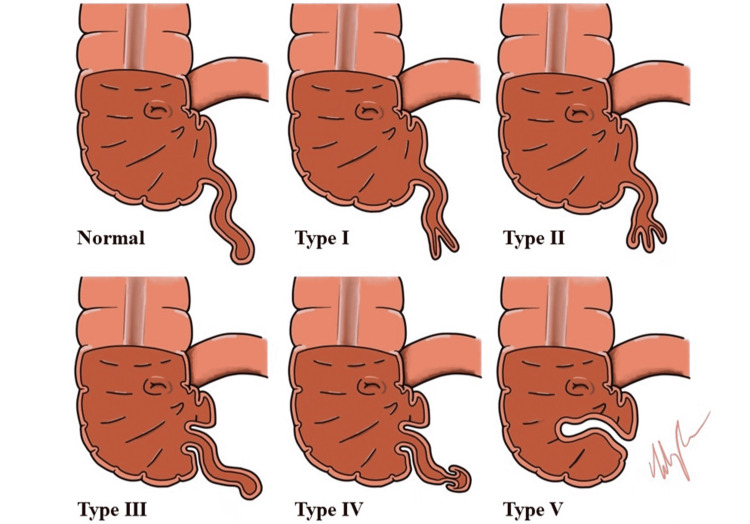
McSwain appendiceal intussusception classification. Type I - mild invagination of the appendiceal tip; Type II - moderate invagination of the appendiceal tip within the proximal appendix; Type III - intussusception of the appendiceal base only; Type IV - a retrograde intussusception; Type V - complete appendiceal intussusception into the cecum. Image drawn by Lily Chen.

In 1941, McSwain described an anatomical classiﬁcation based on the region of the appendix that is intussuscepted [7]. There are ﬁve anatomical classiﬁcations of appendiceal intussusception: Type I - mild invagination of the appendiceal tip; Type II - moderate invagination of the appendiceal tip within the proximal appendix; Type III - intussusception of the appendiceal base only; Type IV - a retrograde intussusception; and Type V - complete appendiceal intussusception into the cecum (Figure 2).

A few cases of ileocecal intussusception and concurrent appendicitis have been described in the medical literature. However, to our knowledge, this is the first report of a Type III appendiceal intussusception and concurrent acute appendicitis. Due to concerns about damaging the already inflamed appendix and/or causing injury to the cecum, traditional approaches to performing a laparoscopic appendectomy were not feasible. Fortunately, in our case, the appendix was partially intussuscepted at the base, and we were able to remove a small portion of the cecum while preserving the ileocecal valve. The optimal surgical approach depends on appendiceal intussusception classification. For example, for those with Type V appendiceal intussusceptions, other surgical options may include ligation of the mesoappendix which will lead to necrosis and sloughing of the fully intussuscepted appendix into the bowel lumen [5].

Typical appendiceal intussusceptions can be either idiopathic or due to predisposing factors such as anatomical variation, duplications, neoplasm, angiodysplasia, fecalith, foreign bodies, lymphoid hyperplasia, or underlying disorders such as cystic fibrosis [8]. It is unclear whether the inflamed appendix acted as a lead point resulting in the intussusception or the intussusception resulted in appendiceal obstruction and inflammation.

## Conclusions

Appendiceal intussusceptions are rare and can also lead to acute appendicitis. They are typically discovered intraoperatively. Here, we present a concurrent Type III appendiceal intussusception with acute appendicitis managed laparoscopically with appendectomy with partial cecectomy. Laparoscopic management is feasible and safe.
